# Mirror therapy for postoperative functional recovery after surgical repair of upper-limb traumatic peripheral nerve injuries: a systematic review and meta-analysis

**DOI:** 10.3389/fneur.2025.1689568

**Published:** 2025-10-14

**Authors:** Jincheng Xu, Dandan Lin, Lina Jian, Wei Liao, Shuming Yang

**Affiliations:** ^1^Department of Rehabilitation Medicine, Affiliated Huishan Hospital of Xinglin College, Nantong University, Wuxi Huishan District People's Hospital, Wuxi, Jiangsu, China; ^2^Department of Pain Rehabilitation, The Fourth Hospital of Hebei Medical University, Shijiazhuang, Hebei, China; ^3^Department of Rehabilitation Medicine, The Fifth Affiliated Hospital of Guangzhou Medical University, Guangzhou, China

**Keywords:** mirror therapy, traumatic peripheral nerve injury, sensory function, hand function, rehabilitation

## Abstract

**Background:**

Traumatic peripheral nerve injuries of the upper limb often lead to substantial motor and sensory deficits, posing significant challenges to functional recovery and quality of life. Mirror therapy, a visually guided neurorehabilitation technique, has shown potential in enhancing upper limb function, yet its effectiveness in traumatic peripheral nerve injuries remains inconclusive.

**Methods:**

This systematic review and meta-analysis followed PRISMA 2020 guidelines. Randomized controlled trials involving adult patients with upper limb traumatic peripheral nerve injuries treated with mirror therapy were identified through searches of seven major databases up to Augst 2025. Methodological quality was assessed using the PEDro scale, and pooled analyses were performed using standard mean differences with 95% confidence intervals.

**Results:**

Seven clinical studies involving 112 participants were included and five randomized controlled trials contributed to the meta-analysis. Mirror therapy significantly improved hand function measured by the Rosen Score (SMD = 0.24; 95% CI: 0.02 to 0.46; *p* = 0.03; I^2^ = 0%). Improvements in grip strength (SMD = 0.45; *p* = 0.26) and sensory outcomes (SWM: SMD = 1.05; *p* = 0.07; 2PD: SMD = 0.45; *p* = 0.26) did not reach statistical significance. Pain-related outcomes were inconsistently reported. Subgroup analysis was not feasible due to intervention heterogeneity and limited sample sizes. Certainty of evidence was moderate for hand function and low to very low for other outcomes.

**Conclusion:**

Mirror therapy may offer modest benefits in hand function recovery following upper limb traumatic peripheral nerve injury. However, current evidence is limited by small sample sizes, methodological heterogeneity, and low study quality. No significant effects were observed for sensory or pain-related outcomes. Further high-quality randomized controlled trials with standardized protocols and long-term follow-up are needed to establish the clinical efficacy and optimize the use of mirror therapy in this population.

**Systematic review registration:**

https://www.crd.york.ac.uk/PROSPERO/view/CRD42023437659.

## 1 Introduction

Traumatic peripheral nerve injury (TPNI) has emerged as an important clinical and public health issue, with its incidence increasing alongside socioeconomic development. Contributing factors include motor vehicle accidents, penetrating trauma, lacerations, gunshot wounds, falls, burns, fractures, ischemia, traction, and crush injuries (1–4). Among all TPNI cases, injuries involving the upper extremities represent a substantial proportion, though prevalence and incidence vary across regions. For example, a 2019 study in the United States reported an incidence of 1.69% for upper limb TPNI (5), while a 2011 study in Iran reported a rate of 1.3% (6). In contrast, a 2022 study from South Korea documented a decline in incidence from 1.07% in 2008 to 0.79% in 2018 (7), suggesting regional and temporal variability. In upper limb TPNI, the radial, ulnar, and median nerves—as well as the brachial plexus—are most frequently involved. Among these, the radial nerve is most commonly affected, followed by the ulnar and median nerves (8). Such injuries are frequently associated with substantial motor and sensory dysfunction, persistent pain, and a decline in overall quality of life (9–11).

MT is a widely recognized rehabilitation technique, effective in promoting motor recovery and alleviating pain in patients with various neurological conditions (12, 13). It uses mirrors to create optical illusions that deceive the brain into perceiving motor and sensory feedback from the affected limbs (14). MT is based on the concept of mirror neurons in the brain, which activate during both action performance and observation (15). Mirror neurons are specialized cells that activate when a person performs an action or observes another performing the same action (16). MT aims to activate mirror neurons and stimulate neural pathways involved in motor control and sensory perception through the use of visual feedback via mirrors (15). In MT, the patient positions a mirror to reflect the unaffected limb, creating the illusion of normal movement in the injured limb. The patient then performs symmetrical movements of both limbs during a series of mirror exercises (17). A systematic review evaluating the effectiveness of MT in improving hand function and sensory recovery in individuals with TPNI is currently lacking. Therefore, a systematic review of the available studies was conducted to assess the effects of MT on these patients.

## 2 Methods

This study was conducted following the PRISMA guidelines (Preferred Reporting Items for Systematic Reviews and Meta-Analyses) and was registered with PROSPERO (registration no. CRD42023437659) (18). Ethical approval was not required.

### 2.1 Search strategy

Databases including PubMed, EMBASE, MEDLINE, the Cochrane Library, WANFANG DATA, CNKI and PEDro were systematically searched from their inception to Augst 5, 2025. Relevant studies examining the efficacy of MT for the treatment of TPNI were identified using specific keywords and controlled vocabulary tailored individually to each database. Detailed search strategies for each database are provided in Appendix 1.

### 2.2 Study selection and data collection

Study selection was carried out in two distinct phases. During the first stage, two reviewers (WL and LNJ) independently screened titles and abstracts based on the predefined eligibility criteria. If relevance could not be ascertained from the abstract alone, the full text was retrieved for further assessment. In the second stage, the same reviewers independently reviewed the full texts to confirm inclusion. Any disagreements were resolved through discussion, and unresolved cases were adjudicated by a third reviewer (DDL). Reference management and duplicate removal were performed using EndNote™ (version 20.5, Clarivate Analytics, Philadelphia, PA) in combination with manual verification.

Data extraction was initially conducted by one reviewer (DDL), and subsequently cross-checked by two independent reviewers (WL and LNJ) to ensure completeness and accuracy. Extracted variables were organized using a customized Microsoft Excel spreadsheet (Microsoft®, United States). Key data included: (i) sample size; (ii) participant demographics and clinical characteristics (e.g., age, sex, condition); and (iii) intervention details such as treatment protocol, dosage, primary outcome measures, evaluation time points, and principal findings. Any discrepancies in the data extraction process were discussed until consensus was reached.

### 2.3 Inclusion and exclusion criteria

This review included the following eligibility criteria: (1) randomized controlled trials (RCTs); (2) adult patients who had undergone upper limb peripheral nerve repair; (3) studies in which MT was the primary intervention. Exclusion criteria included: (1) studies involving pediatric populations (under 18 years of age); (2) studies that did not involve MT or primarily examined other interventions; (3) studies investigating treatments outside the scope of physical or occupational therapy; (4) studies involving patients with comorbidities or additional diagnoses that could confound treatment outcomes.

### 2.4 Quality assessment

The methodological quality of the included studies was assessed using the PEDro Scale, an 11-item, standardized tool specifically designed for evaluating RCTs. This scale provides a comprehensive assessment of key dimensions such as internal validity, quality of reporting, and the interpretability of findings. Of the 11 items, 10 contribute to the final score, yielding a total score range from 0 to 10 (19). In this review, two authors independently conducted the quality assessments for each study. Discrepancies were resolved through discussion until consensus was reached, thereby improving the reliability of the evaluation process.

### 2.5 The risk of bias assessment

Risk of bias was assessed using the original Cochrane Risk of Bias (RoB 1.0) tool, in alignment with the methodological framework outlined in the Cochrane Handbook for Systematic Reviews of Interventions (20). This instrument evaluates five key methodological domains: random sequence generation, deviations from intended interventions, missing outcome data, outcome measurement, and selective outcome reporting. Each domain was rated as having a low risk, high risk, or some concerns, according to predefined judgment criteria. The assessments were independently performed by two reviewers. Any inconsistencies were addressed through discussion, with a third reviewer consulted in cases requiring further resolution to ensure judgment consistency.

## 3 Results

### 3.1 Study selection

**Figure 1:**
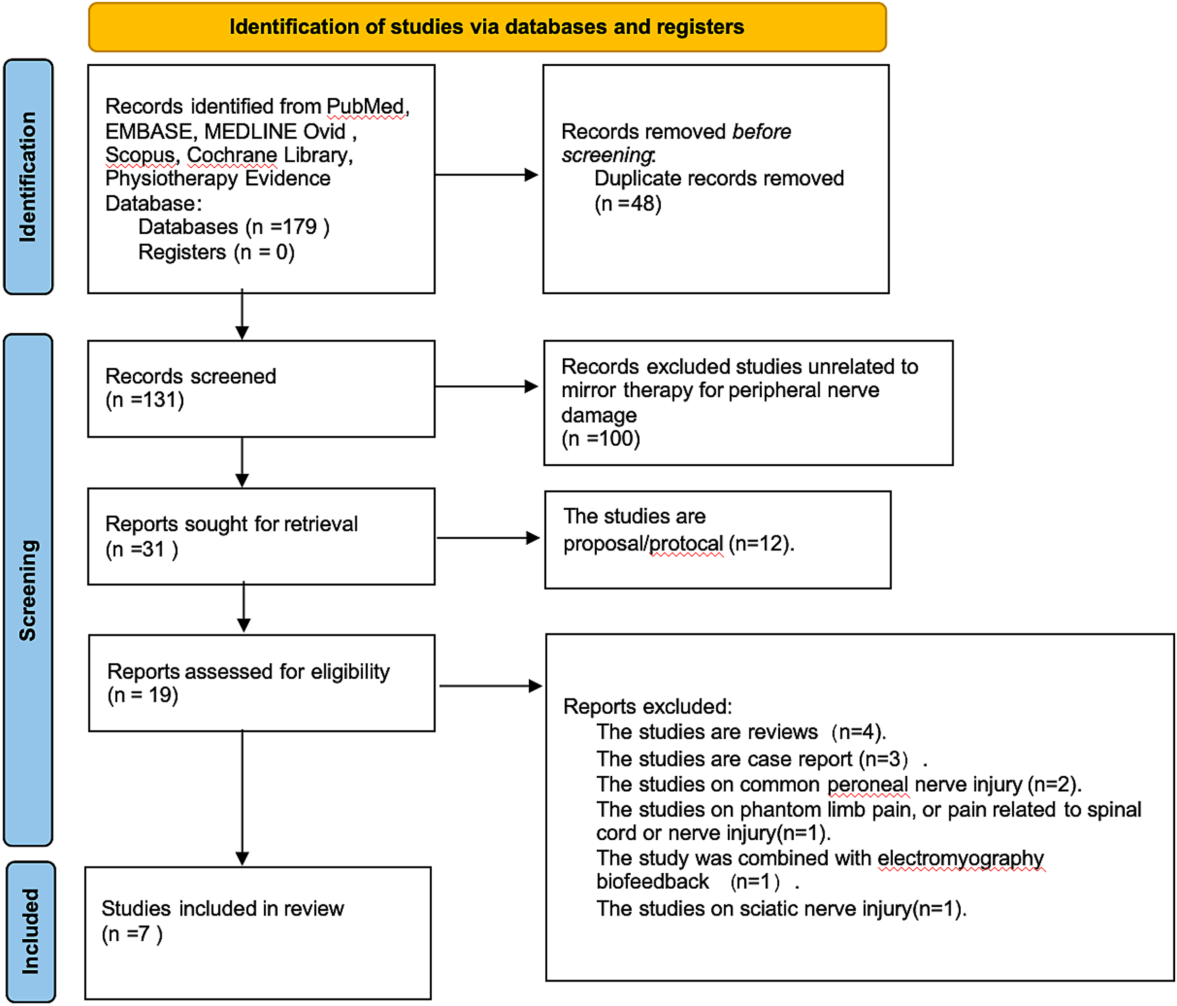
Flowchart of literature screening.

An initial pool of 179 records was retrieved from the selected databases. Following screening and eligibility assessment, seven studies met the inclusion criteria for this review (Figure 1). All included studies were published in English. Two trials were conducted in Taiwan (21, 22). The remaining five comprised trials from Turkey (23), Brazil (24), Iran (25), Sweden (26), and one conducted through a multinational collaboration across the United States, the Netherlands, and Sweden (27). Of the seven studies, five provided sufficient, harmonizable outcome data and were included in the meta-analysis. The other two were not quantitatively synthesized: in one, key outcome data could not be obtained despite attempts to contact the authors, and the other was a 4–9 year follow-up of a previously included trial. To avoid double counting and unit-of-analysis errors, both were included only in the narrative synthesis.

### 3.2 Characteristics of included studies

This review included seven studies (total *n* = 112) evaluating MT for upper-limb TPNI. Because several reports presented outcomes at multiple time points and, in some cases, across more than one publication, methodological quality was appraised at the study level. Sample sizes ranged from 6 to 29 participants. Mean age in intervention arms ranged from 24.25 to 45.70 years and in control arms from 20.50 to 52.70 years. Most injuries involved the median or ulnar nerve. Baseline characteristics (e.g., age) were generally comparable between groups.

For all included studies, we explicitly verified that participants assigned to MT also received the same conventional postoperative rehabilitation as the control group (e.g., routine physiotherapy/occupational therapy) with matched type, frequency, and dose. Accordingly, MT functioned strictly as an adjunct to standard care in every pooled trial, and the meta-analytic estimates therefore reflect the incremental effect of MT beyond conventional rehabilitation. Any deviations or uncertainties regarding co-interventions were recorded as potential sources of bias in the risk-of-bias assessment. For each study, we extracted country, participant demographics, MT delivery, session dose and duration, details of conventional therapy in each arm, comparator type, outcome measures, assessment time points, and main findings. Table 1 provides a structured summary.

**Table 1:** Characteristics of the seven included studies.

Study (year, country)	Sample (I/C)	Age (I/C, yrs)	Injury Type	Duration	Intervention vs Comparator (dose)	Primary outcomes	Assessment time points	Key findings (concise)
Rosen et al., 2015 (USA/NL/SE)	15/14	40/41	Median nerve/ulnar nerve	6 months	I: Usual care + Early home-based MT 4–5×/day, 10 min each; later both groups received sensory re-education 5×/day. C: Usual care → later same re-education.	Rosen score; 2PD; STI	Every 3 months, 2 assessments	MT improved tactile discrimination vs. control, no between-group differences in motor, pain, or total score.
Vikström et al., 2018 (Sweden)	9/11	43 (19–63)/44 (20–69)	Median nerve/ulnar nerve	4–9 years follow-up	Same cohort as Rosen et al., 2015; I: early sensory relearning starting within 1 week post-op; C: traditional rehabilitation starting after sensory perception detected.	Rosen score; 2PD; STI; Grip (Jamar); Q-DASH; CISS	4–9 years post-surgery	Early relearning group maintained superior sensory recovery, dexterity, and self-reported grip/clumsiness/fine motor function compared with controls; no differences in motor or pain domains.(**Note:** 4–9 year long-term follow-up of Rosen et al., 2015 RCT; not treated as an independent trial in meta-analysis.)
Paula et al., 2016 (Brazil)	11/9	24.25/29.25	Median nerve/ulnar nerve	6 months	I: Early sensory re-education from 1 week post-op (30 min × 3/week) + home MT 30 min/day; C: Late sensory re-education starting 3–5 months post-op.	Rosen; SWM; 2PD; STI; Grip (Jamar); Sollerman; Q-DASH	Every 3 months, 2 assessments	At mid-term, MT was not superior to late sensory re-education, though clinical improvements were observed.
Saberi et al., 2018 (Iran)	10/10	29.9/31	Median nerve/ulnar nerve	8 weeks	Both: Conventional rehab 40 min × 3/week; I: + MT 15 min × 5/week (supervised 3/week + home 2/week).	Rosen; SWM; 2PD; STI	Pre- and post-intervention	MT plus sensory re-learning significantly improved superficial sensation and tactile discrimination.
Hsu et al., 2019 (Taiwan)	6/5	35.7/39	Median nerve/ulnar nerve	12 weeks	Both: Hand therapy 20 min + PT 20 min, 3×/week; I: + phased MT 15 min/session.	SWM; Grip (Jamar); MMDT	T1 baseline; T2 post-treatment; T3 12-week follow-up	Greater gains in fine hand function in the MT group, no significant between-group difference in SWM.
Chen et al., 2022 (Taiwan)	3/3	45.7/52.7	Median nerve/ulnar nerve	12 weeks	Both: Conventional PT 60 min × 2/week; I: + afferent–efferent sensorimotor training (MT) 30 min/session (10 min motor + 20 min sensory).	SWM; 2PD; Grip (Jamar); MMDT; Q-DASH	Pre; 12 weeks; 3-month follow-up	Early MT may enhance cortical activation, neuroplasticity patterns differ from traditional sensory re-education.
Kablanoğlu & Sade et al., 2024 (Turkey)	14/12	41/20.5	Peripheral nerve	12 weeks	Both: Standard rehab 45 min × 7/week; I: + MT 15 min × 5/week.	VAS; Q-DASH; SWM; DHI; Grip (Jamar)	Week 5 (pre) and Week 12 (post)	Both groups improved, except DHI, most outcomes favored MT.

This table summarizes key information from six clinical trials evaluating the effects of mirror therapy (MT) on upper limb peripheral nerve injury. Data include study origin, number and demographics of participants, intervention type and duration, treatment dosage, outcome measures, evaluation time points, and study results. 2PD, two-point discrimination; STI, shape texture identification; SWM, Semmes-Weinstein monofilaments; Q-DASH, Quick Disabilities of the Arm, Shoulder and Hand Questionnaire; MMDT, Minnesota Manual Dexterity Test; VAS, visual analog scale; DHI, Duruöz Hand Index.

### 3.3 Evidence quality evaluation

The methodological quality assessment, based on the PEDro scale, is summarized in Table 2. Scores ranged from 3 to 7 points. Accordingly, two studies were classified as high quality (21, 22), four as moderate quality (24–27), and one as low quality (23). This variability highlights methodological heterogeneity, which should be considered when interpreting the findings.

**Table 2:** Risk of bias assessment using the physiotherapy evidence database scale (PEDro).

Study	Eligibility	Item 1	Item 2	Item 3	Item 4	Item 5	Item 6	Item 7	Item 8	Item 9	Item 10	Total score	Quality
B. Rosen et al. (2015)	yes	1	1	0	0	0	0	0	0	1	1	4	fair
Vikström et al.(2018)	yes	1	1	0	0	0	0	0	0	1	1	4	fair
M. H. Paula et al. (2016)	yes	1	1	0	0	0	1	0	0	1	0	4	fair
F. Saberi, L et al. (2018)	yes	1	1	1	0	0	0	0	0	1	0	4	fair
H.-Y. Hsu et al. (2019)	yes	1	1	1	0	0	1	1	0	1	1	7	good
Y.-H. Chen et al. (2022)	yes	1	1	1	1	1	1	0	0	1	0	7	good
S. Kablanoğlu et al. (2024)	yes	1	1	0	0	0	0	0	0	1	0	3	poor

### 3.4 Risk of bias assessment results

**Figure 2:**
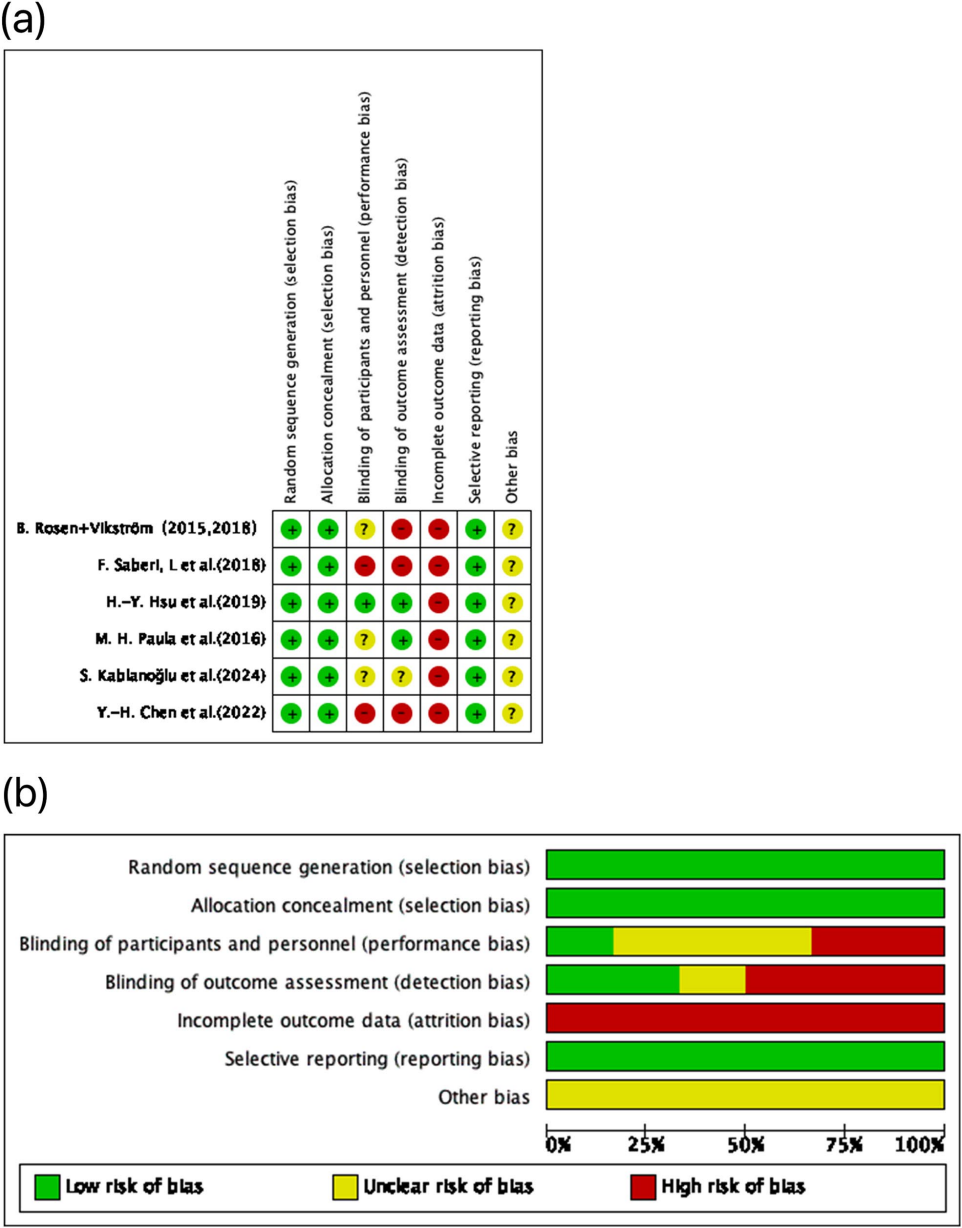
Risk of bias assessment for the included studies using the Cochrane RoB 1.0 tool. **(A)** Domain-level risk of bias ratings for each individual study, covering five key areas of potential methodological bias. **(B)** Aggregated distribution of risk of bias classifications across all included trials. Color coding indicates the risk level: green denotes low risk, yellow indicates some concerns, and red reflects high risk.

Among the seven included studies, one was judged low risk of bias (21), whereas six were judged high risk of bias (22–27). The predominant concerns involved selection bias (item 2), performance bias (item 3), and detection bias (item 4), alongside a frequent problem of >20% missing outcome data without prespecified handling strategies—conditions that can undermine effect estimates and internal validity (Figure 2). Importantly, Rosen et al. (26) and Vikström et al. (27) report on the same cohort, with Vikström representing a *post-hoc* follow-up of the original Rosen trial. Accordingly, these two publications were treated as a single study for the purposes of risk-of-bias assessment. This study lacked assessor blinding and had substantial attrition. Saberi et al. (25) also showed high risk due to participant blinding, assessor blinding, and missing data. Paula et al. (24) and Kablanoğlu et al. (23) were primarily affected by missing data. Although Chen et al. (21) was rated overall low risk, minor concerns remained regarding blinding and attrition, but these did not alter the overall judgment.

### 3.5 Clinical outcomes

#### 3.5.1 Sensory testing

All seven studies assessed sensory function using the Semmes–Weinstein Monofilament (SWM) and two-point discrimination (2PD) tests (21–27). Three trials reported significantly greater improvements in the MT group than in controls (23, 25, 27), whereas three found no significant between group differences (21, 22, 24). At long term follow up (4–9 years after nerve repair), Vikström et al. reported superior performance in the early sensory relearning group on the Rosen sensory domain, particularly in discriminative touch/tactile gnosis and dexterity (26).

#### 3.5.2 Pain testing

Three studies assessed pain (23, 25, 27). One trial showed a greater reduction in visual analog scale (VAS) pain with MT than with control (23), while two found no significant between-group differences (25, 27). Consistent with these findings, Vikström et al. detected no between group differences in the Rosen pain/discomfort domain or in cold sensitivity (CISS) at long-term follow-up (26).

#### 3.5.3 Hand function assessment

Five studies assessed hand function using validated measures (21–24, 27). All five measured grip strength with a Jamar dynamometer; three favored MT (21–23), and two found no significant between-group differences (24, 27). Two studies used the Quick Disabilities of the Arm, Shoulder and Hand Questionnaire (Q-DASH); one favored MT (23) and one reported comparable outcomes (24). Manual dexterity, assessed with the Purdue Pegboard Test and the Minnesota Manual Dexterity Test (MMDT), favored MT in both trials that reported these outcomes (21, 22). One study also found better performance on the Pinch Holding Up Activity test after MT (21), whereas the Duruöz Hand Index (DHI) showed no between-group difference in a single study (23). At the long-term follow up, Vikström et al. found no between-group differences in the Rosen motor domain or in the total Rosen score. However, participants who received early sensory relearning reported fewer problems with grip function, clumsiness, and fine motor tasks, and DASH scores were similar between groups (26).

### 3.6 Meta-analysis

#### 3.6.1 Effect of MT on sensory function compared to control group (measured using SWM and 2PD)

**Figure 3:**
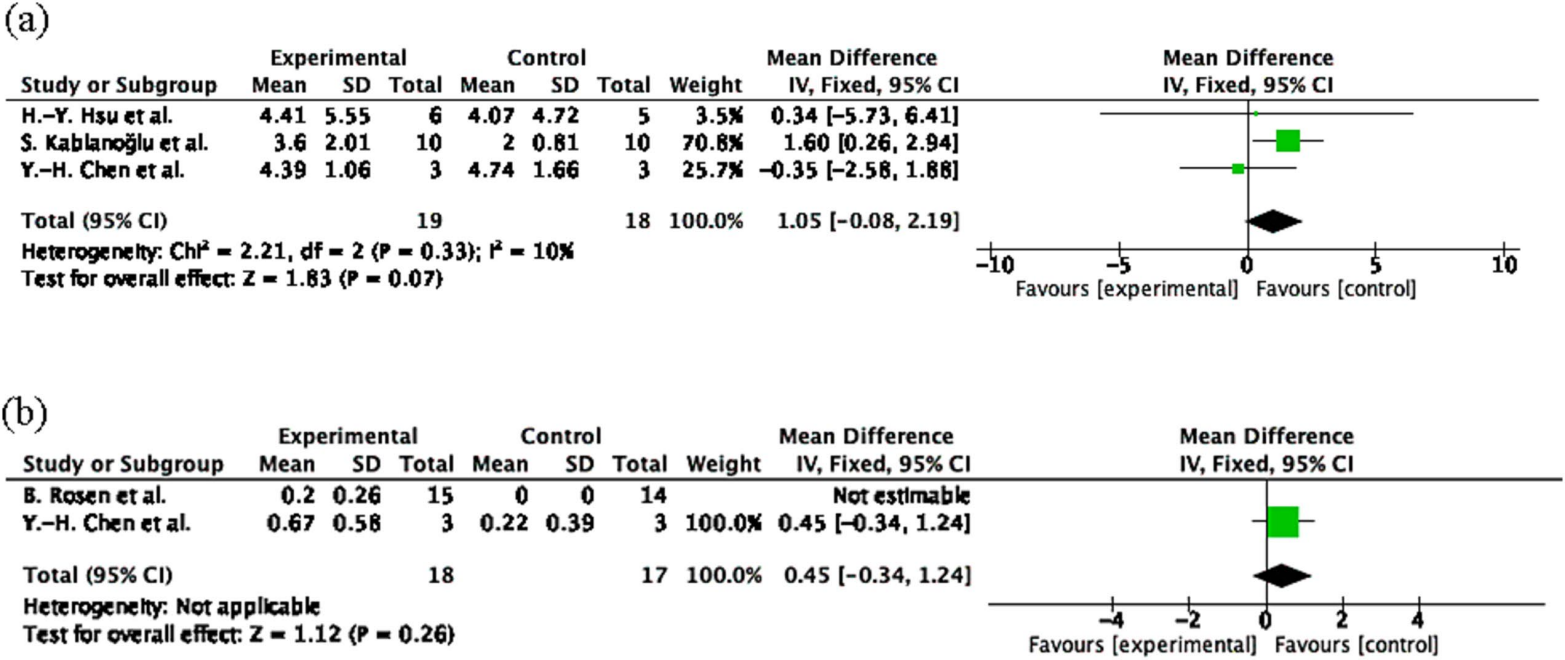
Sensory outcome measures following mirror therapy intervention. **(A)** Comparison of Semmes-Weinstein monofilament (SWM) test results before and after treatment in the mirror therapy and control groups. **(B)** Comparison of two-point discrimination (2PD) before and after treatment in the mirror therapy and control groups. Forest plots illustrate the efficacy of mirror therapy compared to control interventions. Statistical significance was defined as *p* < 0.05.

The pooled effect size for SWM was 1.05 (95% CI: −0.08 to 2.19, *p* = 0.07; Figure 3A), and for 2PD was 0.45 (95% CI: −0.34 to 1.24, *p* = 0.26; Figure 3B), both indicating non-significant effects. Heterogeneity was low for SWM (I^2^ = 10%) and not applicable for 2PD. The small sample sizes (*n* = 18 experimental; *n* = 17 control) may have limited the statistical power, potentially contributing to the non-significant results.

#### 3.6.2 Effect of MT on hand function compared to control group (measured using Q-DASH and Jamar dynamometer)

**Figure 4:**
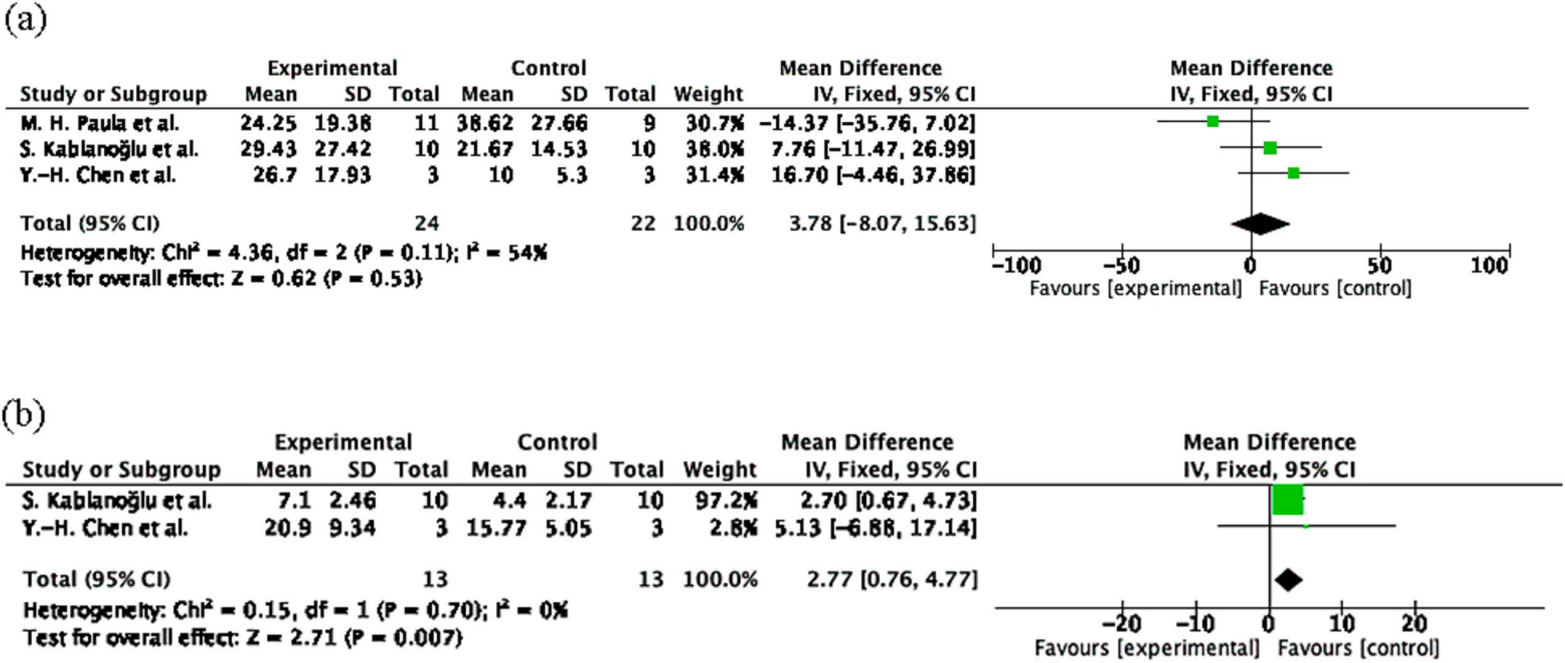
Hand function outcomes following mirror therapy intervention. **(A)** Comparison of Quick Disabilities of the Arm, Shoulder and Hand Questionnaire (Q-DASH) test results before and after treatment in the mirror therapy and control groups. **(B)** Comparison of Jamar dynamometer before and after treatment in the mirror therapy and control groups. Forest plots illustrate the efficacy of mirror therapy compared to control interventions. Statistical significance was defined as *p* < 0.05.

The pooled effect size for Q-DASH was 3.78 (95% CI: −8.07 to 15.63, *p* = 0.53; Figure 4A) and for Jamar Handgrip Strength was 0.45 (95% CI: −0.34 to 1.24, *p* = 0.26; Figure 4B), both indicating non-significant effects. Heterogeneity was moderate for DASH (I^2^ = 54%) and not applicable for Jamar. The limited sample sizes (Q-DASH: *n* = 24 experimental; *n* = 22 control; Jamar: *n* = 18 experimental; *n* = 17 control) and one non-estimable study in the Jamar analysis may have reduced the statistical power.

#### 3.6.3 Effect of MT on overall hand function recovery score compared to the control group (measured using the Rosen score)

**Figure 5:**
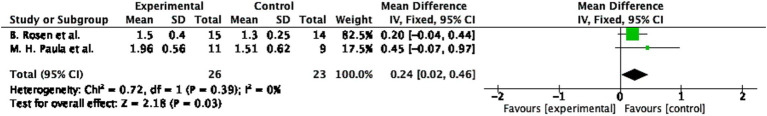
Overall hand function recovery following mirror therapy intervention. Comparison of the Rosen score results before and after treatment in the mirror therapy and control groups. Forest plots illustrate the efficacy of mirror therapy compared to control interventions. Statistical significance was defined as *p* < 0.05.

The pooled effect size for the Rosen Score was 0.24 (95% CI: 0.02 to 0.46, *p* = 0.03; Figure 5), indicating a small but significant improvement in the experimental group. Heterogeneity was low (I^2^ = 0%, *p* = 0.39), suggesting consistent results. The study by Rosen et al. (*n* = 15 experimental; *n* = 14 control) contributed most to the overall effect (82.5%), while Paula et al. (*n* = 11 experimental; *n* = 9 control) had a smaller impact (17.5%). Despite the modest effect size, the narrow CI and absence of heterogeneity enhance result reliability.

### 3.7 Certainty of evidence

Table 3 summarizes the GRADE assessment of evidence certainty. Sensory outcomes (SWM and 2PD) and handgrip strength (Jamar) were rated as low certainty due to risk of bias and imprecision from small sample sizes. The certainty for Q-DASH was very low, reflecting high bias risk, imprecision, and moderate heterogeneity. In contrast, the Rosen score showed moderate certainty, supported by consistent results, narrow confidence intervals, and low heterogeneity despite concerns about blinding.

**Table 3:** Summary of GRADE certainty of evidence for primary and secondary outcomes.

Outcomes (MT vs. control)	Studies and PSS	Risk of bias in studies	Risk of publication bias	Inconsistency	Imprecision	Indirectness	Certainty of evidence
Overall hand function Rosen score Follow-up: mean 12 weeks	2, *n* = 49	Downgrade by one level	Not applicable	No downgrading	No downgrading	No downgrading	Moderate^1^
Sensory function Semmes-Weinstein Monofilaments Follow-up: mean 12 weeks	3, *n* = 37	Downgrade by one level	Not applicable	No downgrading	Downgrade by one level	No downgrading	Low^1^^,^^2^
Sensory function 2 Point Discrimination Follow-up: mean 12 weeks	2, *n* = 35	Downgrade by one level	Not applicable	No downgrading	Downgrade by one level	No downgrading	Low^1^^,^^2^
The strength of handJamar Hand Dynamometer Follow-up: mean 12 weeks	2, *n* = 26	Downgrade by one level	Not applicable	No downgrading	Downgrade by one level	No downgrading	Low^1^^,^^3^
Hand functionthe Quick Disabilities of the Arm, Shoulder, and Hand questionnaire Follow-up: mean 12 weeks	3, *n* = 46	Downgrade by one level	Not applicable	Downgrade by one level	Downgrade by one level	No downgrading	Very Low^1^^,^^4^^,^^5^

This table presents the GRADE (Grading of Recommendations Assessment, Development and Evaluation) assessment of the certainty of evidence for each outcome comparing mirror therapy (MT) and control interventions. Downgrading was based on risk of bias, inconsistency, imprecision, and indirectness. “PSS” refers to pooled sample size. Certainty of evidence was rated as moderate, low, or very low. GRADE, Grading of Recommendations Assessment, Development and Evaluation; MT, Mirror therapy; PSS, pooled sample size.

1 Evaluators and therapists were not blinded and were at risk of bias.

2 The sample size is small, the confidence interval is wide, and the results are imprecise.

3 The sample size was extremely small, the CI crossed the line of invalidity, and the results were significantly imprecise.

4 Moderate heterogeneity (I^2^=54%), considering mild to moderate heterogeneity.

5 The sample size is small, the confidence interval is wide and crosses the invalid line, and there is obvious imprecision.

## 4 Discussion

This systematic review and meta-analysis evaluated the effectiveness of MT for postoperative hand function recovery after upper-limb TPNI. MT is grounded in sensorimotor integration and neuroplasticity (28, 29) and has shown benefits in other neurological populations (12–15), yet the TPNI-specific evidence remains limited in quality and quantity. Of the pooled outcomes, only the Rosen score—an aggregate indicator of overall hand function—showed a statistically significant but small effect (SMD = 0.24; 95% CI, 0.02–0.46; *p* = 0.03) with low heterogeneity (I^2^ = 0%). Pooled results for grip strength and sensory function (SWM and 2PD) were not significant. Pain outcomes were inconsistently reported and could not be meaningfully synthesized. Most trials enrolled small samples (6–29 participants), which reduced statistical power, particularly for sensory and pain endpoints.

Follow-up duration also constrained inference. The trials contributing to the meta-analysis were predominantly short term (≤6 months) (21–24, 27). In contrast, Vikström et al. reported a randomized follow-up at 4–9 years. That study found sustained advantages for early sensory relearning on the Rosen sensory domain, particularly discriminative touch/tactile gnosis and dexterity, and fewer patient-reported problems with grip function, clumsiness, and fine motor tasks. There were no between-group differences in the Rosen motor domain, total Rosen score, Q-DASH, or CISS. Both groups improved from 6 months to long term. Because this follow-up extended a previously included trial, its outcomes were narratively synthesized to avoid double counting (26). These findings suggest that sensory-specific and patient-perceived benefits of early MT can persist even when global motor indices and pain measures do not differ.

The GRADE assessment supports cautious interpretation. Certainty was moderate for overall hand function and low to very low for other outcomes due to risk of bias, imprecision, and inconsistency. Common methodological limitations included unclear randomization, absence of blinding of participants, therapists, and assessors, incomplete outcome reporting, and heterogeneous intervention protocols, consistent with PEDro and Cochrane RoB 1.0 evaluations. Substantial variation in treatment parameters (frequency, duration, task design, and level of supervision) further limited comparability and precluded informative subgroup analyses.

Mechanistic data remain sparse. MT is hypothesized to engage visual feedback pathways and drive experience-dependent plasticity in sensorimotor networks (28, 29). Chen et al. reported increased cortical activation on fMRI immediately after nerve repair in patients receiving MT (22). Future trials should embed imaging or electrophysiological biomarkers to test mechanism and dose–response relationships.

Clinical findings were mixed across individual trials. Saberi et al. reported significant gains in sensory discrimination (25), whereas Hsu et al. (21) and Paula et al. (24) observed no material effects on sensory or pain outcomes. Kablano et al. found no significant differences in grip strength or pain but noted a trend toward better clinical responsiveness with MT (23). Differences in nerve type and lesion level, timing and intensity of MT, outcome instruments, and rehabilitation settings likely contributed to these discrepancies.

In summary, MT may yield modest improvements in overall hand function after TPNI, and early sensory-oriented protocols may confer durable sensory advantages on long-term observation. However, the small number of trials, methodological weaknesses, heterogeneity of interventions, and limited long-term data restrict confidence and generalizability. Future research should prioritize adequately powered randomized trials with standardized, reportable treatment parameters; rigorous bias control (allocation concealment, assessor blinding, prespecified outcomes, and complete data handling); consistent core outcome sets including patient-reported measures; and follow-up beyond 12 months. Work to identify patient-level moderators and to integrate mechanistic assessments (e.g., fMRI/fNIRS, quantitative sensory testing) will help clarify who benefits most and how MT should be optimized in peripheral nerve rehabilitation.

### 4.1 Limitations

This review has several limitations. (1) Small cumulative sample size (*n* = 112 across six RCTs; individual trials 6–29 participants), limiting the precision of pooled estimates, particularly for secondary endpoints such as sensory recovery and pain. (2) Substantial between trial heterogeneity in intervention protocols (duration, session frequency, task content, and degree of therapist involvement), constraining credible subgroup/meta regression analyses and limiting external validity. (3) Methodological limitations and risk of bias, including unclear sequence generation and allocation concealment, limited participant and/or assessor blinding, and incomplete outcome data; domain ratings on Cochrane RoB 1.0 and PEDro scores indicated predominantly “some concerns” to “high” risk. (4) Non-standardized outcome assessment, especially for pain, with inconsistent use of validated measures and objective quantification, hindering cross study comparability and potentially attenuating pooled effect estimates. (5) Short follow up durations (typically immediate post-intervention or early short-term only), precluding firm conclusions about the durability of treatment effects.

## 5 Conclusion

MT may be used as an adjunct to improve hand function after upper-limb TPNI, but current evidence supports only modest functional gains. The literature is based on few, heterogeneous trials with generally low methodological quality. Most pooled outcomes, particularly sensory function and grip strength did not reach statistical significance, and small sample sizes with variable treatment parameters contribute to imprecision and heterogeneity. Follow-up in the RCTs included in the meta-analysis was short. Although a 4–9-year randomized follow-up by Vikström et al. suggested durable sensory advantages with early sensory relearning, those data were narratively synthesized and not pooled, so long-term effects remain uncertain. Clinicians should therefore apply MT cautiously, preferably as part of a multimodal rehabilitation program rather than a stand-alone intervention.

To enable definitive recommendations, future studies should employ adequately powered, high-quality randomized controlled trials with standardized, fully reported intervention protocols (e.g., clearly specified session frequency, duration, and intensity) and prespecified, clinically meaningful outcomes. Rigorous methods are essential, including allocation concealment, blinded outcome assessment, preregistration, and appropriate handling of missing data (e.g., intention-to-treat analyses). Trials should adopt harmonized core outcome sets that include both patient-reported and objective measures, use consistent assessment time points, and extend follow-up to ≥12 months. Identifying patient-level moderators—such as age, nerve involved and lesion level, injury severity, and timing post-surgery—will clarify who benefits most and inform stratified care. Embedding mechanistic assessments (e.g., fMRI/fNIRS, quantitative sensory testing) can elucidate dose–response relationships and neurophysiological pathways, supporting optimized, individualized use of MT in peripheral nerve rehabilitation.

## Data Availability

The original contributions presented in the study are included in the article/Supplementary material, further inquiries can be directed to the corresponding author.
