# Supplementary material for: Mirror therapy for postoperative functional recovery after surgical repair of upper-limb traumatic peripheral nerve injuries: a systematic review and meta-analysis
Source: Front Neurol. 2025 Oct 14;16:1689568. doi: 10.3389/fneur.2025.1689568 (PMC12558756; doi:10.3389/fneur.2025.1689568)
Supplement: Supplementary file 1 [file Table_4.docx]

**Supplementary Appendix 1-Search strategy**

1. **PubMed:**

Query:(((((((Mirror Movement Therapy[Title/Abstract]) OR (Mirror Movement Therapies[Title/Abstract])) OR (Movement Therapies, Mirror[Title/Abstract])) OR (Movement Therapy, Mirror[Title/Abstract])) OR (Therapies, Mirror Movement[Title/Abstract])) OR (Therapy, Mirror Movement[Title/Abstract])) OR (Mirror Therapy[Title/Abstract])) AND ((((Peripheral Nerve Injuries[Title/Abstract]) OR (Peripheral Nerve Injury[Title/Abstract])) OR (Nerve Injuries, Peripheral[Title/Abstract])) OR (Nerve Injury, Peripheral[Title/Abstract]))

Results: 9 Date: 5 Aug 2025

1. **Embase:**

Query:

Session Results

.......................................................

No. Query Results Results Date

#21. #17 AND #20 107 5 Aug 2025

#20. #18 OR #19 59,825 5 Aug 2025

#19. 'peripheral nerve injury'/exp OR 'peripheral 59,825 5 Aug 2025

nerve injury' OR (peripheral AND ('nerve'/exp OR

nerve) AND ('injury'/exp OR injury)) OR

'peripheral nerve damage':ab,ti OR 'peripheral

nerve injuries':ab,ti OR 'peripheral nerve

trauma':ab,ti

#18. 'peripheral nerve injury'/exp 14,772 5 Aug 2025

#17. #14 OR #16 14,124 5 Aug 2025

#16. 'mirror therapy'/exp OR 'mirror therapy' OR 14,124 5 Aug 2025

(('mirror'/exp OR mirror) AND ('therapy'/exp OR

therapy)) OR 'mirror box therapy':ab,ti OR

'mirror feedback therapy':ab,ti OR 'mirror

movement therapy':ab,ti OR ('mirror

therapy':ab,ti AND mt:ab,ti) OR 'mirror visual

feedback':ab,ti OR ('mirror visual

feedback':ab,ti AND mvf:ab,ti AND therapy:ab,ti)

OR 'mirror visual feedback therapy':ab,ti OR

('mirror visual feedback therapy':ab,ti AND

mvft:ab,ti) OR 'mirror visual therapy':ab,ti OR

(mvft:ab,ti AND 'mirror visual feedback

therapy':ab,ti)

#14. 'mirror therapy'/exp 915 5 Aug 2025

.......................................................

1. **Cochrane library:**

Query:

Date: 5 Aug 2025

#1 MeSH descriptor: [Mirror Movement Therapy] explode all trees 36

#2 (Mirror therapy OR Movement Therapy, Mirror OR Movement Therapies, Mirror OR Mirror Therapy OR Mirror Movement Therapies OR Therapies, Mirror Movement OR Therapy, Mirror Movement):ti,ab,kw (Word variations have been searched) 2037

#3 #1 OR #2 2037

#4 MeSH descriptor: [Peripheral Nerve Injuries] explode all trees 145

#5 (Peripheral Nerve Injury OR Nerve Injury, Peripheral OR Nerve Injuries, Peripheral):ti,ab,kw (Word variations have been searched) 1066

#6 #4 OR #5 1066

#7 #3 AND #6 18

1. **MEDLINE:**

Query:

Search: 1: mirror movement therapy (MeSH Heading (No Explode)) Date Run: Tue Aug 05 2025 14:26:00 GMT+0800 (中国标准时间) Results: 73

2: mirror movement therapy (Abstract) OR mirror box therapy (Abstract) OR mirror feedback therapy (Abstract) OR mirror movement therapy (Abstract) OR mirror therapy (MT) (Abstract) OR mirror visual feedback (Abstract) OR mirror visual feedback (MVF) therapy (Abstract) OR mirror visual feedback therapy (Abstract) OR mirror visual feedback therapy (MVFT) (Abstract) OR mirror visual therapy (Abstract) OR MVFT (mirror visual feedback therapy) (Abstract) OR mirror therapy (Abstract)

Date Run: Tue Aug 05 2025 14:29:20 GMT+0800 (中国标准时间) Results: 3981

3: peripheral nerve damage (Abstract) OR peripheral nerve injuries (Abstract) OR peripheral nerve trauma (Abstract) OR peripheral nerve injury (Abstract)

Date Run: Tue Aug 05 2025 14:30:44 GMT+0800 (中国标准时间) Results: 20839

4: #1 or #2

Date Run: Tue Aug 05 2025 14:31:20 GMT+0800 (中国标准时间) Results: 3985

5: peripheral nerve injuries (MeSH Heading (No Explode))

Date Run: Tue Aug 05 2025 14:32:39 GMT+0800 (中国标准时间) Results: 8677

6: #3 or #5

Date Run: Tue Aug 05 2025 14:33:18 GMT+0800 (中国标准时间) Results: 26014

7: #4 and #6

Date Run: Tue Aug 05 2025 14:33:57 GMT+0800 (中国标准时间) Results: 29

1. **PEDro:**

Query:

Mirror therapy* Peripheral nerve injury* Results:3

**6.WANFANG DATA:**

Query:

1."镜像疗法" AND "周围神经损伤" Results: 3

2."镜像治疗" AND ("正中神经损伤" OR "桡神经损伤" OR "尺神经损伤")Results: 0

3."镜像疗法" AND "臂丛神经损伤" Results: 2

4."镜像治疗" AND "康复" AND "周围神经" Results: 1

Date: 5 Aug 2025 Total:6

**7.CNKI:**

Query:

1."镜像疗法" AND "周围神经损伤" Results: 3

2."镜像治疗" AND ("正中神经损伤" OR "桡神经损伤" OR "尺神经损伤")Results: 0

3."镜像疗法" AND "臂丛神经损伤" Results: 3

4."镜像治疗" AND "康复" AND "周围神经" Results: 1

Date: 5 Aug 2025 Total:7
